# Parental incarceration and psychiatric disorders, suicidal behavior, risk‐taking, and substance misuse events in offspring: A longitudinal within‐individual study

**DOI:** 10.1111/jcpp.70152

**Published:** 2026-04-09

**Authors:** Aurora Järvinen, Ralf Kuja‐Halkola, Isabell Brikell, Zheng Chang, Brian M. D'Onofrio, Seena Fazel, Henrik Larsson, Paul Lichtenstein, Antti Latvala

**Affiliations:** ^1^ Department of Medical Epidemiology and Biostatistics Karolinska Institutet Stockholm Sweden; ^2^ Institute of Criminology and Legal Policy University of Helsinki Helsinki Finland; ^3^ Department of Global Public Health and Primary Care University of Bergen Bergen Norway; ^4^ Department of Biomedicine Aarhus University Aarhus Denmark; ^5^ Department of Psychological and Brain Sciences Indiana University Bloomington Indiana USA; ^6^ Department of Psychiatry University of Oxford Oxford UK; ^7^ School of Medical Sciences Örebro University Örebro Sweden

**Keywords:** Parental incarceration, intergenerational relations, psychopathology, behavioral problems, register‐based study, quasi‐experimental study

## Abstract

**Background:**

Parental incarceration (PI) is associated with mental and behavioral problems in offspring, but causality remains unclear. We conducted a quasi‐experimental within‐individual study on the associations of PI with offspring psychiatric disorders, suicidal behavior, risk‐taking, and substance misuse events, aiming to test the effect of PI on offspring.

**Methods:**

The study included 43,011 and 5,912 individuals born in Sweden 1973–2010 and exposed to paternal or maternal incarceration, respectively, between ages 10 and 30. Using data from nationwide registers available until the end of 2020, we examined associations between PI and offspring time‐varying events in a longitudinal within‐individual design to control for time‐invariant confounding. We further studied potential moderation effects in within‐individual models by offspring sex, family living arrangements, age, and other factors. For comparison, we conducted corresponding between‐individual analyses.

**Results:**

Overall, no clear within‐individual associations between paternal or maternal incarceration and offspring events were observed (HRs 0.99–1.08 and 0.82–1.03, respectively), but in a sensitivity analysis, the first recorded exposure to PI was associated with a slightly increased risk of some events (HRs 1.08–1.10). Regarding moderation effects, preliminary evidence suggested that child–father coresiding, paternal offending type, and exposure age slightly moderated the within‐individual associations for certain outcomes. Statistically significant associations were found in all between‐individual models.

**Conclusions:**

The elevated rates of mental and behavioral problems among offspring exposed to PI are unlikely to be due to a direct impact of PI, but an impact may exist under certain circumstances. These findings can help in targeting support to this vulnerable group.

## Introduction

On any given day, millions of children worldwide experience parental incarceration (PI), and the number is growing as incarceration rates continue to rise globally, including the Western world (Clegg, Spitz, Usmani, & Wolcke, 2024). There is also a growing interest in the intergenerational effects of PI, with many studies reporting associations between PI and offspring internalizing problems, self‐harm, externalizing and risk‐taking behavior, and substance misuse (Boch & Ford, 2018; Luk, Hui, Tsang, Fung, & Chan, 2023; Rowell‐Cunsolo, Bellerose, & Borbely, 2024). However, individual studies vary considerably in the strength of these associations, reflecting substantial methodological heterogeneity across studies, such as variation in study populations, definitions, measures, and study designs (Giordano & Copp, 2015; Haskins, Amorim, & Mingo, 2018).

One of the main unresolved issues concerns the nature of the observed associations. Most theoretical accounts assume a causal relationship between PI and offspring outcomes. For example, the risk of internalizing problems and self‐harm has been suggested to be explained by disrupted child–parent attachment after separation from the incarcerated parent, altered neurobiological stress responses following the traumatic loss, social stigma, and reduced family resources leading to increased parental stress, anxiety, and challenges to meet the child's emotional needs (Antle, Gibson, & Krohn, 2020; Beresford, Loucks, & Raikes, 2020; Johnson & Arditti, 2023; Luk et al., 2023). Explanations proposed for offspring externalizing behavior include social learning, reduced parental supervision accompanied with increased time spent with antisocial peers, and the adoption of such behaviors as a coping mechanism (Antle et al., 2020; Johnson & Arditti, 2023; Rowell‐Cunsolo et al., 2024). However, the associations may also be partly or fully explained by other disadvantage and hardship often present in these families. Children experiencing PI are a highly selected group exposed to a range of other adversities, such as parental crime, mental disorders, substance misuse, family economic hardship, domestic violence, and child abuse (Boch, Warren, & Ford, 2019; Giordano & Copp, 2015; Turney, 2018). Also, shared genetic liabilities between parents and children may contribute to elevated risks (Murray, Farrington, & Sekol, 2012).

Accounting for the impact of clustered disadvantage constitutes a central methodological challenge in this area of research (Giordano & Copp, 2015; Haskins et al., 2018; Kirk & Wakefield, 2018). The ability to account for selection effects varies widely across studies, contributing to heterogeneity in their findings. Most existing studies have estimated associations between PI and offspring outcomes by comparing unrelated individuals who differ in their exposure to PI, adjusting for observed covariates to mitigate selection bias. In such studies, PI has typically been associated with offspring outcomes (e.g., Alcalá, Horino, & Delva, 2019; Bravo et al., 2024; Gifford et al., 2019; Le, Deardorff, Lahiff, & Harley, 2019). Some studies have made greater efforts to address causality, for example, by extensively adjusting for between‐group differences in numerous background characteristics and childhood adversities, often observing a reduction in the relative importance of PI in predicting offspring outcomes (e.g., Boch et al., 2019; Turney, 2014). Other studies have used clinic or court samples or comparison groups consisting of other disadvantaged children to better isolate the potential independent effect of PI from that of background factors, often finding attenuated associations (Jackson, Testa, Semenza, & Vaughn, 2021; Murray et al., 2012; Murray, Bijleveljd, Farrington, & Loeber, 2014). A few studies have relied on propensity score matching methods aiming to ensure balanced unexposed and exposed groups by matching individuals on measured background factors. These studies have provided somewhat heterogenous results, with associations attenuating to non‐significance in some studies (e.g., Bradshaw, Hannigan, Creaven, & Muldoon, 2020; Turney & Wildeman, 2015), but remaining significant in others (e.g., Mears & Siennick, 2016; Turney, 2017). While studies that more carefully consider confounding suggest somewhat less consistent associations between PI and offspring health and behavioral outcomes, they have been largely unable to consider selection bias caused by unobserved confounding, such as genetic and unmeasured environmental factors. This limits their ability to fully answer the key question of whether PI is a risk marker or an independent risk factor.

Further, research to date has inadequately considered the varying circumstances in which PI takes place (Haskins et al., 2018; Kirk & Wakefield, 2018). Specifically, the potential effect of PI may vary depending on the specific characteristics of PI, the incarcerated parent, the exposed child, and the wider family context, but evidence is still preliminary and also here causality remains largely unexplored (Boch & Ford, 2018; Gadian et al., 2025; Wildeman, Goldman, & Turney, 2018). For example, offense type and sentence length may be important moderators (Murray et al., 2012; Wildeman et al., 2018), with one study indicating particularly elevated risks among those with a parent incarcerated for nonviolent offenses (Wildeman, 2010). Further, the sex of the child, the child's age at the time of PI, and the family's living arrangements prior to PI may also play a role, with preliminary evidence suggesting particularly elevated risks for male offspring (Geller, Cooper, Garfinkel, Schwartz‐Soicher, & Mincy, 2012; Herreros‐Fraile et al., 2023; Johnson & Arditti, 2023), offspring exposed in middle childhood (ages 7–11) (Herreros‐Fraile et al., 2023), and those who had shared the same household as the incarcerated parent (Geller et al., 2012; Turney & Goldberg, 2019). As survey‐based studies rarely contain all these contextual details, the use of linked administrative data can provide more extensive information on the conditions under which PI takes place (Kirk & Wakefield, 2018; Wildeman et al., 2018).

Aiming to test the possible causal impact of PI, we conducted a longitudinal within‐individual study on the associations between PI and offspring psychiatric disorders, psychotropic medication use, suicidal behavior, risk‐taking, and substance misuse events, examining exposure to paternal, maternal, and any parental incarceration. Based on Swedish population register data with follow‐up from age 10 to age 30, we examined the associations using a quasi‐experimental within‐individual design that accounted for all time‐stable environmental and individual‐level factors, expecting to observe a decrease in the strength of associations under this design. To account for the variety of circumstances in which PI takes place, we also examined several moderating factors for the within‐individual effects. The study complements our previous research on parental offending and its associations with offspring mental, behavioral, and social outcomes, which described elevated risks of many outcomes among children of parents with criminal convictions, with particularly high risks among those whose parents had been sentenced to prison (Järvinen et al., 2024). The current study provides further insight into the unique role of PI and the mechanisms underlying the associations.

## Methods

Data for this non‐preregistered study were obtained from several Swedish nationwide population registers linked by the unique personal identification number available for each person born in Sweden or immigrated to Sweden (Ludvigsson et al., 2016). At the time of the study, linked register data were available until the end of 2020. As Statistics Sweden had pseudonymized all data before making them available to researchers, informed consent was not required. Ethical approval for the register linkage was provided by the Swedish Ethical Review Authority (Dnr 2020‐06540).

### Study cohorts

Using information from the Total Population Register (Ludvigsson et al., 2016) and the Multi‐Generation Register (Ekbom, 2011), we identified individuals born in Sweden between January 1, 1973, and December 31, 2010, and linked them to their biological parents. With data from the Sanction Register of the Swedish Prison and Probation Service (SPPS), we restricted the sample to those exposed to paternal or maternal incarceration at some point between the ages of 10 and 30 between January 1, 2003, and December 31, 2020. This yielded a cohort of 43,011 individuals with an incarcerated father (*N* = 22,552), 5,912 individuals with an incarcerated mother  (*N* = 2,996), and 47,798 individuals with either parent incarcerated (*N* = 25,548). Parental incarceration was examined from 2003 to 2020, as the sanction register contains data on prison periods with an end date on January 1, 2003, or later. In secondary between‐individual analyses, we used a population‐based cohort (for details, see Appendix S1). The study cohorts were further modified for some analyses, described in detail in Appendix S1.

### Measures

#### Exposure

We defined paternal/maternal incarceration as a time‐varying binary variable indicating any paternal or maternal custodial sentence served in prison or another closed institution according to the sanction register of SPPS. Besides analyzing paternal and maternal incarceration separately, we also examined exposure to any parental incarceration. We excluded prison periods shorter than 14 days, in accordance with the SPPS's definition of prison sentences, which range from 14 days to life imprisonment (Kriminalvården, n.d.). We examined exposure between the ages of 10 and 30. The earliest age of exposure was set at 10 years to maximize the use of data while taking into account that the studied outcomes are not typically diagnosed before late childhood. The latest age of exposure was set at 30 years as the potential effect of PI was assumed to be most detectable by early adulthood, when direct contact with the childhood family is usually greater than later in life. In within‐individual models, the time‐varying binary exposure denoted periods of PI and periods without PI during follow‐up, including all prison periods that occurred between the child's ages of 10 and 30. In between‐individual models, parent's first prison sentence during follow‐up was treated as a time‐varying exposure.

#### Outcomes

We examined five outcome categories: (1) psychiatric disorders (including diagnoses of all psychiatric disorders except substance use disorders), (2) psychotropic medication use, (3) suicidal behavior, (4) risk‐taking behaviors (e.g., diagnoses of injuries, poisonings, and accidents, and convictions for selected traffic offenses), and (5) substance misuse events (including diagnoses of substance use disorders and convictions for driving under the influence). Data on the events were derived from the National Patient Register, the Prescribed Drug Register, and the National Crime Register, described in detail in Appendix S1 and Table S1. We examined events from age 10 onwards, except for the categories of substance misuse and risk‐taking behavior, which were studied from age 15 onwards, because they included criminal convictions that can be imposed at age 15 at the earliest (the age of legal responsibility in Sweden).

#### Stratifying variables

We examined potential moderation effects with stratified analyses across four variables: (1) offspring legally registered sex from the Total Population Register (Ludvigsson et al., 2016), (2) a binary variable indicating whether the child and their father had lived in the same neighborhood for at least 8 years of the child's first 10 years of life, based on the Demographic Statistical Areas classification (described in more detail in Appendix S1) (Statistics Sweden, n.d.), (3) a binary variable indicating whether the father had convictions of violent (including sexual) offenses during the child's first 10 years of life, according to the National Crime Register (convictions described in Table S2) (Brottsförebyggande rådet, n.d.), and (4) prison sentence length, with each sentence classified by its duration as either less than 1 year or at least 1 year. In addition, we examined moderation by age by studying the interaction between age and exposure to PI (see below).

### Statistical analysis

#### Main analyses

We applied a within‐individual design as our primary analytical approach. The design compares the rate of each outcome event during periods of exposure to PI with periods without such exposure within the same individual. As each individual acts as their own control, the design implicitly accounts for all time‐invariant confounders, regardless of whether they are observed or unobserved, that is, factors that are constant within an individual across time, such as genetic background and early life experiences (Allison, 2009), thus allowing for the examination of the possible direct impact of PI. To allow for comparison of outcome risk within an individual between periods when exposed versus unexposed to PI, each exposed individual must have at least one period during the follow‐up when the parent was not incarcerated.

We fitted stratified Cox regression models treating each individual as a unique stratum (for details, see Appendix S1). This resulted in a separate hazard function for each individual, estimating the rate of time‐to‐event outcome between the periods when the child was exposed versus unexposed to paternal/maternal/either parent's incarceration. All parental prison periods during follow‐up contributed to the estimation, irrespective of their number. Hazard ratios (HRs) estimated ratio of the rate of each event between periods with and without exposure to PI.

To examine whether the within‐individual associations differed according to potential moderators, we further conducted analyses stratified by each of the variables described above. Due to the low number of maternal prison periods, we only studied paternal incarceration in stratified analyses. Further, to examine potential age‐specific effects, we fitted a natural cubic spline with four degrees of freedom for age at exposure, main effect, and interaction with exposure, and then estimated the effect over age in within‐individual analyses (for details, see Appendix S1).

All within‐individual models were adjusted for attained age (as a continuous variable, measured at the start of time span) at each time span. In all models, we clustered the standard errors at the individual level.

#### Secondary analyses

Alongside the within‐individual analyses, we also performed standard between‐individual Cox regression analyses for comparison, aiming to show how the associations manifest themselves when applying a more commonly used approach, accounting only for measured confounders. The models estimated the relative risk of each event during the follow‐up among offspring exposed to paternal/maternal/either parent's incarceration as compared to unexposed offspring (for a detailed description, see Appendix S1). We also conducted between‐individual analyses stratified by each of the factors described above.

#### Sensitivity analyses

We conducted several sensitivity analyses to test the robustness of our findings, using paternal incarceration as exposure. First, to study potential carry‐over effects of the first recorded exposure, we compared the risk of each event during the 1‐year period before the first exposure with the first exposure period and the 1‐year post‐exposure period. Second, we tested whether the results differed when we excluded the first time span (time from study entry to the first event) from the analysis. This was because the underlying time scale was measured differently for the first time span compared with subsequent spans (i.e., time from study entry rather than time since last event). Third, to test the suitability of the within‐individual design for the outcome categories used, we restricted the main analysis to diagnoses that were received during unplanned health care visits, thus excluding diagnoses from routine follow‐up care. The aim was to more accurately capture acute changes in symptoms. Diagnoses with missing information on the type of visit were excluded. Finally, we tested how different age adjustments influenced the within‐individual results by comparing models adjusted for age as a linear effect versus using a natural cubic spline with three degrees of freedom.

We used 95% confidence intervals to estimate the precision of the estimates in all analyses. All analyses were performed using Stata, version 18 (StataCorp, 2023).

## Results

The number of children with an incarcerated father was 43,011 (1.1%), with an average of 1.7 paternal prison sentences per child and an average duration of 7.7 months per sentence. The number of children with an incarcerated mother was 5,912 (0.15%), with an average of 1.6 maternal prison sentences per child and an average duration of 5.5 months per sentence. Descriptive characteristics of children exposed and unexposed to PI are presented in Table 1. The cumulative incidence of each outcome by age 30 by PI status is presented in Table S3. The incidences were highest among those exposed to paternal or maternal incarceration, being 46.3% and 51.8% for psychiatric disorders, 11.0% and 14.9% for suicidal behavior, 70.2% and 73.0% for risk‐taking behavior, and 25.4% and 33.1% for substance misuse events, respectively.

**Table 1:** Characteristics of children born between 1973 and 2010 with neither parent in prison (*N* = 3,872,932), a father in prison (*N* = 43,011), a mother in prison (*N* = 5,912), or either parent in prison (*N* = 47,798) between the ages of 10 and 30 during the period 2003–2020

	Children without parental incarceration (*N* = 3,827,932)	Children with an incarcerated father (*N* = 43,011)	Children with an incarcerated mother (*N* = 5,912)	Children with either parent incarcerated (*N* = 47,798)
Sex, *N* (%)				
Male	1,967,649 (51.4)	22,186 (51.6)	3,054 (51.7)	24,656 (51.6)
Female	1,860,283 (48.6)	20,825 (48.4)	2,858 (48.3)	23,142 (48.4)
Birth year, median (1st and 3rd quartiles)	1991 (1982, 2001)	1992 (1987, 1997)	1991 (1986, 1997)	1992 (1987, 1997)
Father's birth year, median (1st and 3rd quartiles)	1960 (1951, 1968)	1963 (1957, 1969)	1962 (1956, 1968)	1963 (1957, 1969)
Mother's birth year, median (1st and 3rd quartiles)	1963 (1954, 1971)	1965 (1960, 1972)	1966 (1960, 1971)	1965 (1960, 1972)
Paternal immigration status, *N* (%)				
Born in Sweden	3,218,852 (84.1)	30,053 (69.9)	4,499 (76.1)	33,796 (70.7)
Born outside Sweden	608,447 (15.9)	12,945 (30.1)	1,409 (23.8)	13,985 (29.3)
Missing data	633 (0.02)	13 (0.03)	4 (0.1)	17 (0.04)
Maternal immigration status, *N* (%)				
Born in Sweden	3,241,440 (84.7)	32,804 (76.3)	4,659 (78.8)	36,648 (76.7)
Born outside Sweden	586,264 (15.3)	10,204 (23.7)	1,254 (21.2)	11,147 (23.3)
Missing data	228 (0.01)	3 (0.01)	0 (0.0)	3 (0.01)
Highest parental education, *N* (%)				
Primary and lower secondary education	194,550 (5.1)	5,296 (12.3)	1,106 (18.7)	6,062 (12.7)
Upper secondary education	1,624,708 (42.4)	26,592 (61.8)	3,736 (63.2)	29,660 (62.1)
Post‐secondary education	1,891,210 (49.4)	10,918 (25.4)	1,409 (17.7)	11,851 (24.8)
Postgraduate education	101,560 (2.7)	183 (0.4)	20 (0.3)	203 (0.4)
Missing data	15,904 (0.4)	22 (0.1)	1 (0.02)	22 (0.05)
Any paternal psychiatric disorder, *N* (%)	658,988 (17.2)	29,820 (69.3)	2,866 (48.5)	31,827 (66.6)
Any maternal psychiatric disorder, *N* (%)	772,494 (20.2)	18,227 (42.4)	5,042 (85.3)	22,318 (46.7)
Father–child coresiding^a^, *N* (%)				
Father coresiding with the child	2,346,189 (79.7)	12,730 (32.8)	1,853 (34.8)	14,358 (33.3)
Missing data	12,196 (0.4)	135 (0.4)	3,450 (64.7)	157 (0.4)
Mother–child coresiding^a^, *N* (%)				
Mother coresiding with the child	2,774,786 (94.3)	35,061 (90.3)	3,601 (67.6)	37,977 (88.1)
Missing data	12,196 (0.4)	135 (0.4)	28 (0.5)	157 (0.4)
Paternal violent convictions during the child's first 10 years of life, *N* (%)	107,765 (2.8)	16,789 (39.0)	1,606 (27.2)	17,805 (37.3)
Maternal violent convictions during the child's first 10 years of life, *N* (%)	13,558 (0.4)	1,485 (3.5)	1,018 (17.2)	2,214 (4.6)
*N* of parental prison sentences				
Mean (*SD*)	–	1.7 (1.6)	1.6 (1.4)	1.7 (1.7)
Median (1st and 3rd quartiles)	–	1 (1, 2)	1 (1, 2)	1 (1, 2)
Length of prison sentence (months)				
Mean (*SD*)	–	7.7 (14.6)	5.5 (10.5)	7.5 (14.2)
Median (1st and 3rd quartiles)	–	2.8 (1.3, 8.1)	2 (1, 5.5)	2.7 (1.3, 7.9)
Parent's age (years) at the beginning of the first prison sentence				
Mean (*SD*)	–	46.2 (8.1)	44.1 (7.1)	–
Median (1st and 3rd quartiles)	–	45.9 (40.4, 51.5)	44.2 (39.3, 48.8)	–
Offspring's age (years) at the beginning of the first prison sentence				
Mean (*SD*)	–	17.0 (5.8)	18.3 (5.7)	17.1 (5.8)
Median (1st and 3rd quartiles)	–	16.0 (12.0, 21.3)	17.9 (13.4, 22.8)	16.2 (12.1, 21.5)

^a^ Indicates whether the child had lived in the same area as his/her father or mother for at least 8 years of the first 10 years of life. Individuals born in Sweden between 1982 and 2010 (*N* = 2,986,900) were included.

**Figure 1:**
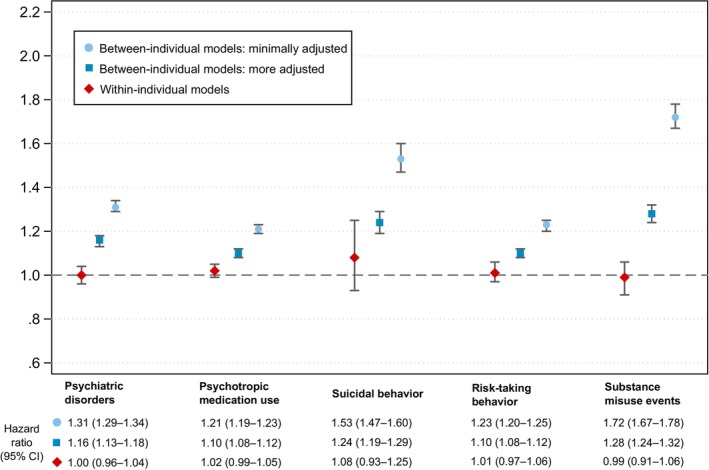
Associations between paternal incarceration and offspring psychiatric disorders, suicidal behavior, risk‐taking, and substance misuse events using within‐individual and between‐individual designs

**Figure 2:**
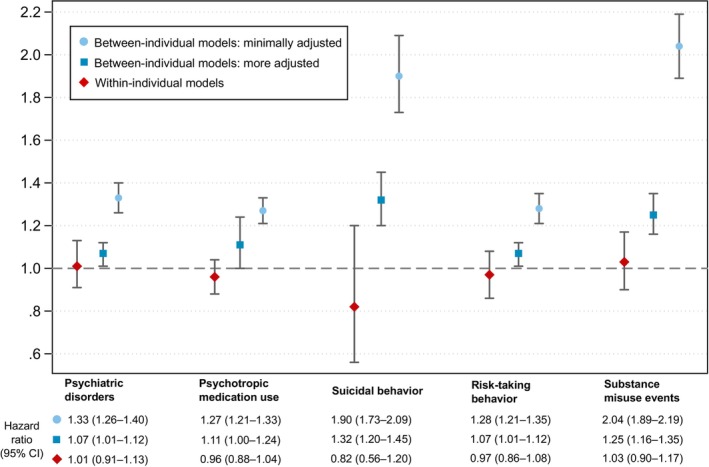
Associations between maternal incarceration and offspring psychiatric disorders, suicidal behavior, risk‐taking, and substance misuse events using within‐individual and between‐individual designs

Figure 1 shows results from the within‐individual Cox regression models for the associations between paternal incarceration and offspring events, accompanied with results from corresponding between‐individual models. Consistently across all outcomes, no statistically significant within‐individual effects were found (HRs ranging from 0.99 to 1.08). By contrast, in between‐individual models, the risk of each event was elevated among the exposed offspring relative to the unexposed group (HRs 1.21–1.72 in partially adjusted models and 1.10–1.28 in more adjusted models). Similar patterns were observed for maternal incarceration (Figure 2): within‐individual associations were statistically nonsignificant (HRs 0.82–1.03), while between‐individual associations reached statistical significance (HRs 1.27–2.04 in partially adjusted models and 1.07–1.32 in more adjusted models). The results were highly similar for any PI (Figure S1).

**Figure 3:**
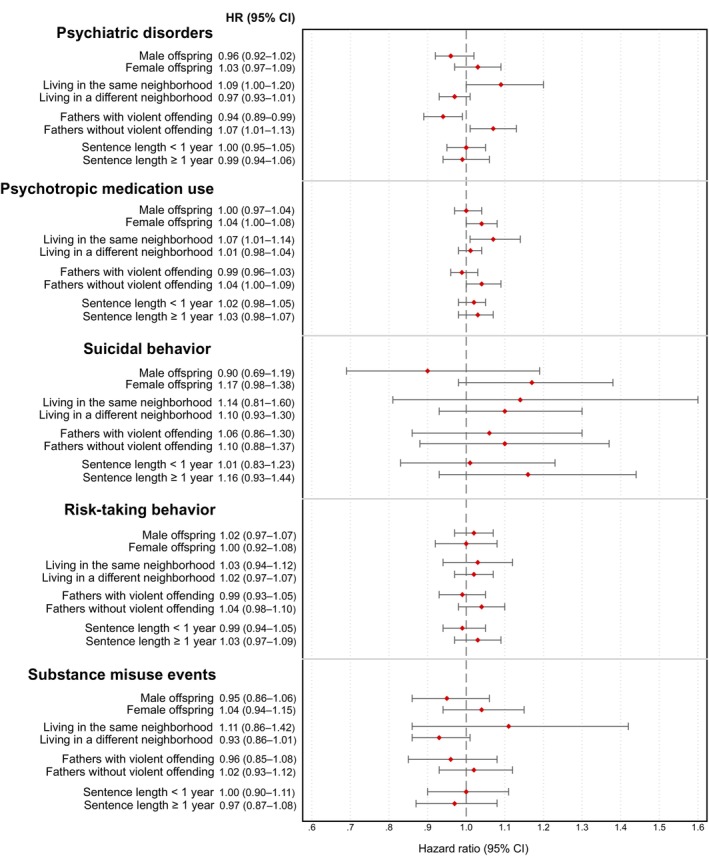
Within‐individual associations between paternal incarceration and offspring mental health and behavioral events, stratified by offspring sex, child–father coresiding, paternal offending type, and sentence length

Figure 3 presents results from within‐individual models stratified by offspring sex, child–father coresiding, paternal offending type, and sentence length. The associations between paternal incarceration and the outcome events were generally statistically nonsignificant for both male (HRs 0.90–1.02) and female (HRs 1.00–1.17) offspring, with no clear sex differences observed. In models stratified by child–father coresiding, paternal incarceration was associated with a slightly increased risk for psychiatric disorders (HR 1.09 [95% CI, 1.00–1.20]) and psychotropic medication use (HR 1.07 [95% CI, 1.01–1.14]) among those who had mostly lived in the same neighborhood as their father. In models stratified by paternal offending type, paternal incarceration was associated with a small increase in the risk of psychiatric disorders and psychotropic medication use (HRs 1.07 [95% CI, 1.01–1.13] and 1.04 [95% CI, 1.00–1.09], respectively) among offspring whose fathers had no history of violent offending. In models stratified by sentence length, no statistically significant within‐individual associations were observed either for short sentences (HRs 0.99–1.02) or long sentences (HRs 0.97–1.16). Results from stratified between‐individual models are given in Table S4. Associations were statistically significant across all models, but some differences were observed: HRs tended to be higher for those with more coresiding and for those whose father had no history of violent offending.

**Figure 4:**
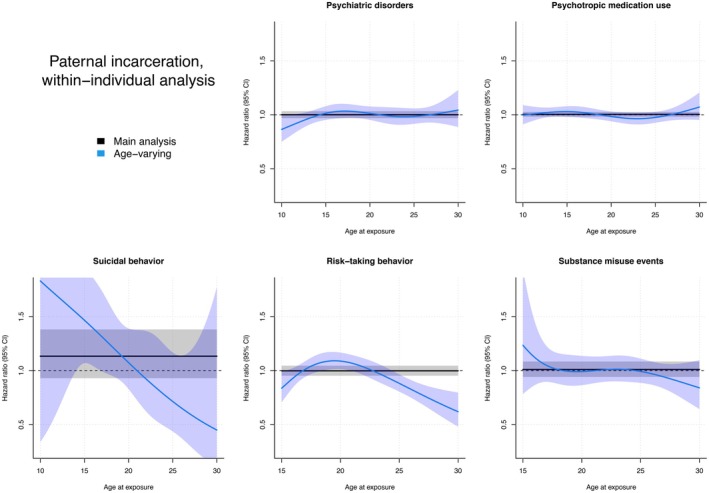
Age‐specific within‐individual associations between paternal incarceration and offspring mental health and behavioral events

In analyses estimating effects of PI over age (Figure 4), no clear age‐specific effects were observed for offspring psychiatric disorders, psychotropic medication use, and substance misuse events. Point estimates for the association between PI and offspring suicidal behavior suggested some age‐dependent variation but were statistically nonsignificant. The association with offspring risk‐taking behavior varied slightly by age, with hazard ratios indicating a modestly elevated risk in early adulthood and a reduced risk thereafter.

In the sensitivity analysis examining potential carry‐over effects, we found that the first recorded exposure to PI during the follow‐up was statistically significantly associated with elevated rates of psychiatric disorders, psychotropic medication use, and risk‐taking behaviors compared with the pre‐exposure period (HRs 1.08–1.10; Table S5). Rates during the post‐exposure period, limited to a maximum of 1 year, did not differ or were reduced compared with the pre‐exposure period. In the analysis excluding the first time span (from study entry to the first event; Table S6), we found very similar estimates as in the main analysis. In the analysis restricted to diagnoses set during unplanned health care visits (Table S7), we found no clear differences in the estimates compared with the main analysis, although the results were less precise. We also found that the results of within‐individual analyses were highly similar regardless of the type of age adjustment (Table S8).

## Discussion

We examined the effects of PI on offspring psychiatric disorders, suicidal and other risk‐taking behaviors (including accidents), and substance misuse events between ages 10 and 30 using Swedish nationwide register data. We used a quasi‐experimental within‐individual design that controls for all time‐invariant confounders within a person. We further conducted stratified analyses and interaction analyses to investigate potential differences in the associations between male and female offspring, by family living arrangements, age, and other factors.

As expected, our study identified offspring exposed to PI as a high‐risk group for adverse mental health and behavioral outcomes. For example, approximately half of the exposed offspring had been diagnosed with a psychiatric disorder, 25%–33% had a record of substance misuse, and 11%–15% had engaged in suicidal behavior by age 30. Despite the notable absolute risks, no clear evidence of a direct effect of PI was found in the main analysis. This was consistent across paternal, maternal, and any parental incarceration. While associations between PI and offspring internalizing and externalizing problems, psychotropic medication use, self‐harm, risk‐taking behaviors, and drug use have been repeatedly observed (e.g., Alcalá et al., 2019; Cumming et al., 2023; Davis & Shlafer, 2017; Luk et al., 2023; Rowell‐Cunsolo et al., 2024), findings are somewhat less consistent in studies that more carefully consider confounding factors (e.g., Boch et al., 2019; Jackson et al., 2021; Murray et al., 2012; Turney & Wildeman, 2015), suggesting that the associations are at least partly explained by other adversity clustered in families. Importantly, however, most studies have been unable to examine the role of selection effects comprehensively. One exception is an Australian longitudinal survey‐based study that examined the within‐individual association between PI and young people's mental health, finding no evidence of a direct effect of PI (Besemer, van de Weijer, & Dennison, 2018). Our study supports and extends this finding by showing no clear within‐individual associations between PI and a broader range of offspring mental health and behavioral outcomes in register data.

However, results from the sensitivity analysis tentatively suggest that the first exposure to PI may differ qualitatively from subsequent exposures. Specifically, the first recorded exposure was associated with a slightly elevated risk of some events as compared to the pre‐exposure period. As the risks did not remain elevated thereafter, the first exposure does not seem to have a lasting carry‐over effect. Nevertheless, the results may indicate that the potential effect of PI varies dynamically according to exposure features that cannot be detected by simply treating PI as a binary variable, but we emphasize the preliminary nature of these findings. As the follow‐up started at age 10 at the earliest and the starting time varied across individuals, we are unlikely to have captured the actual first exposure to PI. Further research with a more nuanced investigation of exposure to PI with consideration of its temporal and sequential patterns is needed.

While our study cannot exclude the possibility that the potential effect of PI depends on dynamics that we were unable to properly capture, the fact that the point estimates were close to unity in all analyses suggests that the potential immediate impact of PI, leading to changes in the mental health or behavioral status in offspring during the period when the parent is incarcerated, is, in any case, very modest. We thus argue that the commonly‐employed causal explanations, particularly those emphasizing the immediate effect of parental absence due to incarceration, are unlikely to be the primary explanatory models for the elevated rates of mental health and behavioral problems among offspring exposed to PI. Instead, we suggest that the main explanation is selection effects, likely due to both stable environmental stressors and genetic influences accumulated in these families (Murray & Farrington, 2008; Rhodes et al., 2023). The contrast between the within‐individual and between‐individual results illustrates how simply comparing differently exposed individuals and adjusting for measured covariates may lead to erroneous conclusions about the nature of the associations, emphasizing the importance of research designs capable of accounting for unobserved confounding.

The results from stratified analyses generally supported the conclusions of the main analyses but leave open the possibility of a weak immediate effect of PI under certain circumstances. We found tentative evidence of a modest effect of paternal incarceration on psychiatric conditions among offspring who had mostly lived in the same neighborhood as their father and among those whose fathers had no history of violent offending. Previous studies have similarly suggested a possible moderating effect of these factors (Assaraf & Factor, 2025; Geller et al., 2012; Turney & Wildeman, 2013; Wildeman, 2010). These children are more likely to maintain a personal relationship with their father, and paternal incarceration in this context may exert an independent effect on their mental health beyond the influence of other family adversity. However, as these findings emerged from multiple exploratory analyses and may not hold after correction for multiple testing, they should be interpreted with caution and warrant replication.

We found no clear evidence for the moderating role of offspring sex. Some previous studies have found that male offspring are often at higher risk (Geller et al., 2012; Johnson & Arditti, 2023; Wildeman et al., 2018). Our within‐individual findings suggest, however, that the previously observed sex differences are unlikely to be primarily explained by males and females reacting differently to PI per se. Similarly, neither short nor long sentences were associated with the outcome events, suggesting that the risk of these events does not depend on the duration of PI. As for moderation by exposure age, some age‐dependent variation in the magnitude of the association between PI and offspring risk‐taking and suicidal behavior was found, which may indicate age‐specific effects. However, given the considerable uncertainty of the estimates, we consider these findings preliminary and highlight the need for further research.

Overall, our findings suggest that the observed mental health and behavioral problems of children of incarcerated parents are not limited to periods of PI but are more widespread. This underscores the importance of long‐term and comprehensive support for these children and their families, extending beyond periods of PI and, ideally, reaching them before PI takes place. Public health efforts could benefit from shifting the emphasis toward preventive measures that aim to alter harmful behavioral and developmental trajectories in both children and their parents earlier in the life course (e.g., Fazel, Burghart, Wolf, Whiting, & Yu, 2024). As public authorities in Sweden currently lack standardized procedures for identifying or supporting these families (Grönqvist, Niknami, Palme, & Priks, 2025), there is a strong need to develop systematic protocols and interventions to ensure that the needs of this vulnerable group are adequately addressed.

We recognize that our findings are highly context‐specific and leave open the possibility that PI may have a more direct impact in other countries. In the Swedish context, several factors may attenuate the effects of PI on offspring. For example, the Swedish universal health care system (Ludvigsson et al., 2025) may reduce health‐related inequalities between socioeconomic groups, and publicly funded financial support mechanisms can alleviate the economic burden on families experiencing PI. Also, the focus of incarceration on social rehabilitation and minimizing recidivism rather than on punishment (Humphreys, 2023) may attenuate the stigma associated with PI in Sweden, compared to countries with more punitive attitudes. Short prison sentences and family‐friendly prison practices (Sharratt, 2014; Smith, 2015) may also lessen children's perceived burden of PI. Finally, the low prison rates in Sweden may reflect a stronger selection of more disadvantaged people in the prison population, and the incarceration of such a parent may even serve as a protective factor from the perspective of their children.

The results of our study need to be considered in light of several limitations. First, while the within‐individual design controls for all time‐invariant confounding, it cannot account for unmeasured time‐varying factors. It is thus possible that our findings have been influenced by, for example, other family disruptions and life events occurring near the time of exposure to PI. Our results also do not exclude the possibility that unmeasured contextual events preceding PI (e.g., arrest and trial) have an impact. Further, the within‐individual design is limited to evaluating only immediate effects of PI. We were thus unable to answer whether PI has an effect on symptoms and disorders with delayed onset. Second, the within‐individual design assumes independence between exposures to PI. In the sensitivity analysis, we found no clear evidence of carry‐over effects from the first recorded exposure. However, due to data limitations, we were unable to assess whether the actual first lifetime exposure has a distinct, lasting impact. For example, early life exposure may lead to an elevated risk of health problems before the child reaches age 10, and it may also initiate a cascade of adversities that ultimately manifests as symptoms later in life (Turney, 2022). Third, as data on outcomes were largely based on diagnoses made in specialized health care, the study was unable to examine the effects of PI on symptoms or behaviors treated in primary care, school health care, private clinics, or nonprofit organizations, or on individuals who did not seek treatment. This is an important limitation given that most mental health patients are treated in primary care in Sweden (Sundquist, Ohlsson, Sundquist, & Kendler, 2017), and a substantial proportion of self‐injurious behavior does not lead to official records (Hawton, Saunders, & O'Connor, 2012; Madge et al., 2008). However, our analyses of offspring psychotropic medication use, which covered prescriptions issued in both primary and specialized health care, yielded findings highly similar to those of offspring psychiatric disorders, suggesting no significant bias in the results. Also, the timing of events was derived from diagnosis dates, which may not accurately capture the actual onset of symptoms, as there may be a delay between their appearance, admission to health care, and receiving a diagnosis. We addressed this in the sensitivity analysis focusing on unplanned health care visits, and the results showed little difference compared to those of the main analysis. Fourth, the low number of maternal prison periods prevented us from conducting stratified analyses for maternal incarceration. Although no clear differences were found between exposure to maternal versus paternal incarceration in the main analyses, we cannot rule out the possibility that more detailed analyses might yield different results. Fifth, we lacked data on child protection services and foster care placements, which would have provided a valuable addition given the overrepresentation of children exposed to PI in these contexts (Jackson, Testa, & Vaughn, 2023). Finally, as discussed previously, we emphasize the context‐specific nature of our findings and highlight the need for caution in interpreting them in an international context.

## Conclusion

Taken together, this study makes an important contribution to the evidence base on the effects of PI on offspring mental health and behavior by employing a quasi‐experimental study design. We identify children exposed to PI as a vulnerable group at increased risk of adverse psychiatric and behavioral conditions, emphasizing the need for targeted support and health monitoring. As we did not find clear evidence for the elevated rates being attributable to the immediate impact of PI, we suggest that support should be broad‐based and widely available to these families, rather than limited to situations when families are acutely experiencing PI.

## Ethical consideration

Ethical approval for the register linkage was provided by the Swedish Ethical Review Authority on 7 January 2021 (reference number 2020–06540). Informed consent is not required for pseudonymized register‐based studies in Sweden.

**Key points**

**What is known?**

- Paternal incarceration has been associated with poor mental health and behavioral problems in offspring, but it remains unclear whether these links are causal in nature.

**What is new?**

- This quasi‐experimental within‐individual study examined the effect of parental incarceration on offspring psychiatric disorders, psychotropic medication use, suicidal behavior, risk‐taking, and substance misuse events.
- The elevated rates of psychiatric, behavioral, and substance use problems among offspring exposed to parental incarceration are unlikely to be due to a direct impact of parental incarceration, but rather to selection effects. However, preliminary evidence suggests that a modest impact may exist under certain circumstances.

**What is relevant?**

- The findings can help in designing and targeting support to this vulnerable group.

## Supporting information


**Appendix S1.** Supporting methods.
**Table S1.** Offspring psychiatric and behavioral events with corresponding ICD‐10 codes, ATC codes, or convictions.
**Table S2.** Convictions for violent and sexual offenses.
**Table S3.** Cumulative incidence of psychiatric disorders, suicidal behavior, risk‐taking, and substance misuse events by age 30 in children with neither parent in prison, father in prison, mother in prison, or either parent in prison.
**Figure S1.** Associations between parental incarceration and offspring psychiatric disorders, suicidal behavior, risk‐taking, and substance misuse events using within‐individual and between‐individual designs.
**Table S4.** Population‐level associations between paternal incarceration and offspring psychiatric and behavioral events, stratified by offspring sex, child–father coresiding, paternal offending type, and sentence length.
**Table S5.** Cox regression models comparing the risk of offspring events during and after the first exposure to paternal incarceration, relative to the pre‐exposure period.
**Table S6.** Associations between paternal incarceration and offspring psychiatric and behavioral events using within‐individual design, with the first time span for each individual included versus excluded from the analysis.
**Table S7.** Associations between paternal incarceration and offspring psychiatric and behavioral events, with analysis restricted to diagnoses received during unplanned health care visits.
**Table S8.** Paternal incarceration and offspring psychiatric and behavioral events using within‐individual design with different age adjustments.

## Data Availability

Due to Swedish data protection laws and ethical regulations, the data cannot be made publicly available. Researchers interested in accessing the data can apply for access from the register holders.

## References

[jcpp70152-bib-0001] Alcalá, H.E. , Horino, M. , & Delva, J. (2019). Psychotropic medication use in children: What role does child adversity play? Social Work Research, 43, 81–89.

[jcpp70152-bib-0002] Allison, P. (2009). Fixed effects regression models. Thousand Oaks, CA: Sage.

[jcpp70152-bib-0003] Antle, K. , Gibson, C.L. , & Krohn, M.D. (2020). The mediating role of family dynamics in the relationship between paternal incarceration and child behavior problems. Journal of Crime and Justice, 43, 16–35.

[jcpp70152-bib-0004] Assaraf, N. , & Factor, R. (2025). Delinquency among children of incarcerated parents: The role of pre‐incarceration parental attachment. Journal of Criminal Justice, 99, 102456.

[jcpp70152-bib-0005] Beresford, S. , Loucks, N. , & Raikes, B. (2020). The health impact on children affected by parental imprisonment. BMJ Paediatrics Open, 4, e000275. 32154384 10.1136/bmjpo-2018-000275 PMC7047477

[jcpp70152-bib-0006] Besemer, K.L. , van de Weijer, S.G.A. , & Dennison, S.M. (2018). Risk marker or risk mechanism? The effect of family, household, and parental imprisonment on children and adults' social support and mental health. Criminal Justice and Behavior, 45, 1154–1173.

[jcpp70152-bib-0007] Boch, S.J. , & Ford, J.L. (2018). Health outcomes of youth in the United States exposed to parental incarceration: An integrative review. Journal of Forensic Nursing, 14, 61–71. 29781966 10.1097/JFN.0000000000000201

[jcpp70152-bib-0008] Boch, S.J. , Warren, B.J. , & Ford, J.L. (2019). Attention, externalizing, and internalizing problems of youth exposed to parental incarceration. Issues in Mental Health Nursing, 40, 466–475. 30958077 10.1080/01612840.2019.1565872 PMC6557679

[jcpp70152-bib-0009] Bradshaw, D. , Hannigan, A. , Creaven, A.M. , & Muldoon, O.T. (2020). Longitudinal associations between parental incarceration and children's emotional and behavioural development: Results from a population cohort study. Child: Care, Health and Development, 46, 195–202. 31810111 10.1111/cch.12732

[jcpp70152-bib-0010] Bravo, L.G. , Meza, J. , Schiff, S.J. , Ahmed, C. , Elliot, T. , La Charite, J. , & Choi, K. (2024). Parental legal system involvement, positive childhood experiences, and suicide risk. Pediatrics, 153, e2023062566. 38779781 10.1542/peds.2023-062566 PMC11153318

[jcpp70152-bib-0011] Brottsförebyggande rådet . (2025). Swedish crime statistics. https://bra.se/bra‐in‐english/home.html

[jcpp70152-bib-0012] Clegg, J. , Spitz, S. , Usmani, A. , & Wolcke, A. (2024). Punishment in modern societies: The prevalence and causes of incarceration around the world. Annual Review of Criminology, 7, 211–231.

[jcpp70152-bib-0013] Cumming, C. , Bell, M.F. , Segal, L. , Spittal, M.J. , Kinner, S.A. , Dennison, S. , … & Preen, D.B. (2023). Maternal incarceration increases the risk of self‐harm but not suicide: A matched cohort study. Epidemiology and Psychiatric Sciences, 32, e33. 37161898 10.1017/S2045796023000264 PMC10227533

[jcpp70152-bib-0014] Davis, L. , & Shlafer, R.J. (2017). Mental health of adolescents with currently and formerly incarcerated parents. Journal of Adolescence, 54, 120–134. 28011442 10.1016/j.adolescence.2016.10.006 PMC5549675

[jcpp70152-bib-0015] Ekbom, A. (2011). The Swedish multi‐generation register. Methods in Molecular Biology, 675, 215–220. 20949391 10.1007/978-1-59745-423-0_10

[jcpp70152-bib-0016] Fazel, S. , Burghart, M. , Wolf, A. , Whiting, D. , & Yu, R. (2024). Effectiveness of violence prevention interventions: Umbrella review of research in the general population. Trauma, Violence & Abuse, 25, 1709–1718. 10.1177/15248380231195880 PMC10913357 37650521

[jcpp70152-bib-0017] Gadian, N. , Dunn, A. , Arundel, D. , Morgan, S. , Harriott, P. , Wainwright, L. , & Plugge, E. (2025). The impact of maternal versus paternal imprisonment on their children's health: A scoping review. PLoS One, 20, e0329131. 40729394 10.1371/journal.pone.0329131 PMC12306776

[jcpp70152-bib-0019] Geller, A. , Cooper, C.E. , Garfinkel, I. , Schwartz‐Soicher, O. , & Mincy, R.B. (2012). Beyond absenteeism: Father incarceration and child development. Demography, 49, 49–76. 22203452 10.1007/s13524-011-0081-9 PMC3703506

[jcpp70152-bib-0020] Gifford, E.J. , Kozecke, L.E. , Golonka, M. , Hill, S.N. , Costello, E.J. , Shanahan, L. , & Copeland, W.E. (2019). Association of parental incarceration with psychiatric and functional outcomes of young adults. JAMA Network Open, 2, e1910005. 31441942 10.1001/jamanetworkopen.2019.10005 PMC6714027

[jcpp70152-bib-0021] Giordano, P.C. , & Copp, J.E. (2015). “packages” of risk implications for determining the effect of maternal incarceration on child wellbeing. Criminology & Public Policy, 14, 157–168. 26617473 10.1111/1745-9133.12118 PMC4657872

[jcpp70152-bib-0022] Grönqvist, H. , Niknami, S. , Palme, M. , & Priks, M. (2025). The intergenerational effects of parental incarceration. The Economic Journal, ueaf136.

[jcpp70152-bib-0023] Haskins, A.R. , Amorim, M. , & Mingo, M. (2018). Parental incarceration and child outcomes: Those at risk, evidence of impacts, methodological insights, and areas of future work. Sociology Compass, 12, e12562.

[jcpp70152-bib-0024] Hawton, K. , Saunders, K.E.A. , & O'Connor, R.C. (2012). Self‐harm and suicide in adolescents. Lancet, 379, 2373–2382. 22726518 10.1016/S0140-6736(12)60322-5

[jcpp70152-bib-0025] Herreros‐Fraile, A. , Carcedo, R.J. , Viedma, A. , Ramos‐Barbero, V. , Fernández‐Rouco, N. , Gomiz‐Pascual, P. , & del Val, C. (2023). Parental incarceration, development, and well‐being: A developmental systematic review. International Journal of Environmental Research and Public Health, 20, 3143. 36833841 10.3390/ijerph20043143 PMC9967200

[jcpp70152-bib-0026] Humphreys, B. (2023). The Nordic carceral system: Examining Scandinavian penal exceptionalism. Nordic Review of International Studies, 2, 72–83.

[jcpp70152-bib-0027] Jackson, D.B. , Testa, A. , Semenza, D.C. , & Vaughn, M.G. (2021). Parental incarceration, child adversity, and child health: A strategic comparison approach. International Journal of Environmental Research and Public Health, 18, 3384. 33805850 10.3390/ijerph18073384 PMC8036687

[jcpp70152-bib-0028] Jackson, D.B. , Testa, A. , & Vaughn, M.G. (2023). Parental incarceration and children's living arrangements in the United States. Child and Adolescent Social Work Journal, 40, 695–711.

[jcpp70152-bib-0029] Järvinen, A. , Lichtenstein, P. , D'Onofrio, B.M. , Fazel, S. , Kuja‐Halkola, R. , & Latvala, A. (2024). Health, behavior, and social outcomes among offspring of parents with criminal convictions: A register‐based study from Sweden. Journal of Child Psychology and Psychiatry, 65, 1590–1600. 38736394 10.1111/jcpp.14003

[jcpp70152-bib-0030] Johnson, E.I. , & Arditti, J.A. (2023). Risk and resilience among children with incarcerated parents: A review and critical reframing. Annual Review of Clinical Psychology, 19, 437–460. 10.1146/annurev-clinpsy-080921-081447 36750264

[jcpp70152-bib-0031] Kirk, D.S. , & Wakefield, S. (2018). Collateral consequences of punishment: A critical review and path forward. Annual Review of Criminology, 1, 171–194.

[jcpp70152-bib-0032] Kriminalvården . (2025). Fängelse, frivård och häkte . https://www.kriminalvarden.se/fangelse‐frivard‐och‐hakte/fangelse/

[jcpp70152-bib-0033] Le, G.T. , Deardorff, J. , Lahiff, M. , & Harley, K.G. (2019). Intergenerational associations between parental incarceration and children's sexual risk taking in young adulthood. Journal of Adolescent Health, 64, 398–404. 10.1016/j.jadohealth.2018.09.028 30514651

[jcpp70152-bib-0034] Ludvigsson, J.F. , Almqvist, C. , Bonamy, A.K.E. , Ljung, R. , Michaëlsson, K. , Neovius, M. , … & Ye, W.M. (2016). Registers of the Swedish total population and their use in medical research. European Journal of Epidemiology, 31, 125–136. 26769609 10.1007/s10654-016-0117-y

[jcpp70152-bib-0036] Ludvigsson, J.F. , Bergman, D. , Lundgren, C.I. , Sundquist, K. , Geijerstam, J.‐L.a. , Glenngård, A.H. , … & Yao, J. (2025). The healthcare system in Sweden. European Journal of Epidemiology, 40, 1–17. 40383868 10.1007/s10654-025-01226-9 PMC12170770

[jcpp70152-bib-0037] Luk, M.S.K. , Hui, C. , Tsang, S.K.M. , Fung, Y.L. , & Chan, C.H.Y. (2023). Physical and psychosocial impacts of parental incarceration on children and adolescents: A systematic review differentiating age of exposure. Adolescent Research Review, 8, 159–178.

[jcpp70152-bib-0038] Madge, N. , Hewitt, A. , Hawton, K. , Wilde, E.J.d. , Corcoran, P. , Fekete, S. , … & Ystgaard, M. (2008). Deliberate self‐harm within an international community sample of young people: Comparative findings from the Child & Adolescent Self‐harm in Europe (CASE) study. Journal of Child Psychology and Psychiatry, 49, 667–677. 18341543 10.1111/j.1469-7610.2008.01879.x

[jcpp70152-bib-0039] Mears, D.P. , & Siennick, S.E. (2016). Young adult outcomes and the life‐course penalties of parental incarceration. Journal of Research in Crime and Delinquency, 53, 3–35.

[jcpp70152-bib-0040] Murray, J. , Bijleveljd, C.C.J.H. , Farrington, D.P. , & Loeber, R. (2014). Effects of parental incarceration on children: Cross‐national comparative studies. Washington, DC: American Psychological Association.

[jcpp70152-bib-0041] Murray, J. , & Farrington, D.P. (2008). The effects of parental imprisonment on children. Crime and Justice, 37, 133–206.

[jcpp70152-bib-0042] Murray, J. , Farrington, D.P. , & Sekol, I. (2012). Children's antisocial behavior, mental health, drug use, and educational performance after parental incarceration: A systematic review and meta‐analysis. Psychological Bulletin, 138, 175–210. 22229730 10.1037/a0026407 PMC3283435

[jcpp70152-bib-0043] Rhodes, C.A. , Thomas, N. , O'Hara, K.L. , Hita, L. , Blake, A. , Wolchik, S.A. , … & Berkel, C. (2023). Enhancing the focus: How does parental incarceration fit into the overall picture of adverse childhood experiences (ACEs) and positive childhood experiences (PCEs)? Research on Child and Adolescent Psychopathology, 51, 1933–1944. 37875642 10.1007/s10802-023-01142-0 PMC11008286

[jcpp70152-bib-0044] Rowell‐Cunsolo, T.L. , Bellerose, M. , & Borbely, C. (2024). Exposure to parental incarceration and subsequent illicit drug use: A review of the literature. Children & Society, 38, 97–115.

[jcpp70152-bib-0045] Sharratt, K. (2014). Children's experiences of contact with imprisoned parents: A comparison between four European countries. European Journal of Criminology, 11, 760–775.

[jcpp70152-bib-0046] Smith, P.S. (2015). Children of imprisoned parents in Scandinavia: Their problems, treatment and the role of Scandinavian penal culture. Law Context: A Socio‐Legal J., 32, 147.

[jcpp70152-bib-0047] StataCorp . (2023). Stata statistical software: Release 18. College Station, TX: StataCorp LLC.

[jcpp70152-bib-0048] Statistics Sweden . (2026). DeSO – Demografiska statistikområden . https://www.scb.se/hitta‐statistik/regional‐statistik‐och‐kartor/regionala‐indelningar/demografiska‐statistikomraden‐deso/

[jcpp70152-bib-0049] Sundquist, J. , Ohlsson, H. , Sundquist, K. , & Kendler, K.S. (2017). Common adult psychiatric disorders in Swedish primary care where most mental health patients are treated. BMC Psychiatry, 17, 235. 28666429 10.1186/s12888-017-1381-4 PMC5493066

[jcpp70152-bib-0050] Turney, K. (2014). Stress proliferation across generations? Examining the relationship between parental incarceration and childhood health. Journal of Health and Social Behavior, 55, 302–319. 25138199 10.1177/0022146514544173

[jcpp70152-bib-0051] Turney, K. (2017). The unequal consequences of mass incarceration for children. Demography, 54, 361–389. 28063011 10.1007/s13524-016-0543-1

[jcpp70152-bib-0052] Turney, K. (2018). Adverse childhood experiences among children of incarcerated parents. Children and Youth Services Review, 89, 218–225.

[jcpp70152-bib-0053] Turney, K. (2022). Chains of adversity: The time‐varying consequences of paternal incarceration for adolescent behavior. Journal of Quantitative Criminology, 38, 159–196.

[jcpp70152-bib-0054] Turney, K. , & Goldberg, R.E. (2019). Paternal incarceration and early sexual onset among adolescents. Population Research and Policy Review, 38, 95–123. 38264735 10.1007/s11113-018-9502-4 PMC10805465

[jcpp70152-bib-0055] Turney, K. , & Wildeman, C. (2013). Redefining relationships: Explaining the countervailing consequences of paternal incarceration for parenting. American Sociological Review, 78, 949–979. 41960306 10.1177/0003122413505589 PMC13061399

[jcpp70152-bib-0056] Turney, K. , & Wildeman, C. (2015). Detrimental for some? Heterogeneous effects of maternal incarceration on child wellbeing. Criminology & Public Policy, 14, 125–156. 38046473 10.1111/1745-9133.12109 PMC10691851

[jcpp70152-bib-0058] Wildeman, C. (2010). Paternal incarceration and children's physically aggressive behaviors evidence from the fragile families and child wellbeing study. Social Forces, 89, 285–309.

[jcpp70152-bib-0059] Wildeman, C. , Goldman, A.W. , & Turney, K. (2018). Parental incarceration and child health in the United States. Epidemiologic Reviews, 40, 146–156. 29635444 10.1093/epirev/mxx013

