# Supplementary material for: Parental incarceration and psychiatric disorders, suicidal behavior, risk‐taking, and substance misuse events in offspring: A longitudinal within‐individual study
Source: J Child Psychol Psychiatry. 2026 Apr 9;67(9):1476–87. doi: 10.1111/jcpp.70152 (PMC13450286; doi:10.1111/jcpp.70152)
Supplement: Supplementary file 1 [file JCPP-67-1476-s001.docx]

**Parental incarceration and psychiatric disorders, suicidal behavior, risk-taking, and substance misuse events in offspring: A longitudinal within-individual study**

**Supporting Information**

**Table of Contents**

[**Appendix S1.** Supporting Methods.	1](#_Toc223033248)

[**Table S1.** Offspring psychiatric and behavioral events with corresponding ICD-10 codes, ATC codes, or convictions.	5](#_Toc223033249)

[**Table S2.** Convictions for violent and sexual offenses	7](#_Toc223033250)

[**Table S3.** Cumulative incidence of psychiatric disorders, suicidal behavior, risk-taking, and substance misuse events by age 30 in children with neither parent in prison, father in prison, mother in prison, or either parent in prison	7](#_Toc223033251)

[**Figure S1.** Associations between parental incarceration and offspring psychiatric disorders, suicidal behavior, risk-taking, and substance misuse events using within-individual and between-individual designs.	8](#_Toc223033252)

[**Table S4.** Population-level associations between paternal incarceration and offspring psychiatric and behavioral events, stratified by offspring sex, child-father coresiding, paternal offending type, and sentence length.	9](#_Toc223033253)

[**Table S5.** Cox regression models comparing the risk of offspring events during and after the first exposure to paternal incarceration, relative to the pre-exposure period.	10](#_Toc223033254)

[**Table S6.** Associations between paternal incarceration and offspring psychiatric and behavioral events using within-individual design, with the first time span for each individual included vs. excluded from the analysis.	10](#_Toc223033255)

[**Table S7.** Associations between paternal incarceration and offspring psychiatric and behavioral events, with analysis restricted to diagnoses received during unplanned health care visits.	11](#_Toc223033256)

[**Table S8.** Paternal incarceration and offspring psychiatric and behavioral events using within-individual design with different age adjustments.	11](#_Toc223033257)

[**References**	12](#_Toc223033258)

# **Appendix S1.** Supporting Methods.

**Study cohort**

***Study cohort for between-individual analyses.*** For comparative between-individual analyses, we used a population-based cohort with the same birth year restrictions as in the within-individual analyses, including all individuals born in Sweden between 1973 and 2010. We excluded those with missing information on father’s or mother’s identity (*N* = 52,540), yielding a sample of 3,875,730 individuals and their parents (1,954,501 fathers and 1,960,018 mothers).

***Study cohort adjustments****.* We made modifications to the original study cohorts for certain analyses. First, as the event categories of substance misuse events and risk-taking behavior included data not only on diagnoses but also on criminal convictions, for which data were only available from age 15 onwards (the age of legal responsibility in Sweden), the cohort was defined to ensure that all birth cohorts had reached at least 15 years of age at the start of follow-up, which was in December 2020 at the latest. For within-individual analyses, we thus included individuals born in Sweden between January 1, 1973 and December 31, 2005 and exposed to PI at some point between the ages of 15 to 30 years during the period January 1, 2003, and December 31, 2020. This yielded samples of 30,586 individuals with an incarcerated father (*N* = 17,085), 4,537 individuals with an incarcerated mother (*N* = 2,467), and 34,437 individuals with either parent incarcerated (*N* = 19,552). For between-individual analyses, the sample included 3,328,442 individuals born in Sweden between 1973 and 2005 and their parents (1,700,575 fathers and 1,701,459 mothers).

Second, we refined the original cohort for analyses of offspring psychotropic medication use, as data on prescribed drugs were only available from 2005 onwards. The study cohort for within-individual analyses thus included individuals born in Sweden between January 1, 1975, and December 31, 2010 and exposed to PI between the ages of 10 to 30 years during the time period from January 1, 2005, to December 31, 2020. This resulted in samples of 38,203 individuals with an incarcerated father (*N* = 20,070), 5,350 individuals with an incarcerated mother (*N* = 2,703), and 42,602 individuals with either parent incarcerated (*N* = 22,773). The corresponding population-level sample comprised 3,658,659 individuals and their parents (1,854,916 fathers and 1,858,372 mothers).

Third, in analyses stratified by information on the child’s and father’s coresiding, the earliest birth cohort included individuals born in 1982, as residential data were available from 1982 onwards (Statistics Sweden, 2026). The study sample for within-individual analyses thus comprised 38,834 individuals (21,105 fathers) born in Sweden between 1982 and 2010 and exposed to PI between the ages of 10 to 30 years during the time period from 2003 to 2020. The corresponding population-level sample comprised 2,986,900 individuals (1,552,063 fathers). As for analyses of offspring substance misuse events and risk-taking behavior stratified by child-father coresiding, we further restricted the sample to individuals born in Sweden between 1982 and 2005. This resulted in a study sample of 26,411 individuals (15,497 fathers) for within-individual analyses and a sample of 2,439,612 individuals (1,297,750 fathers) for population-level analyses.

**Measures**

***Outcomes.*** Data on the outcome events were derived from the National Patient Register, the Prescribed Drug Register, and the National Crime Register. The National Patient Register contains diagnoses set in inpatient and outpatient specialized public health care in Sweden since 1973 and 2001, respectively, based on the International Classification of Diseases (ICD) (Ludvigsson et al., 2011). Diagnoses based on ICD-10 (1997–present) were collected. The Prescribed Drug Register includes all prescribed drugs according to the Anatomical Therapeutic Chemical (ATC) classification, dispensed at pharmacies in Sweden from July 2005 onwards (Wallerstedt et al., 2016). The National Crime Register covers all district court level convictions nationwide since 1973, including information on the timing, type of offense, and the sanction (Brottsförebyggande rådet, 2025). The event categories with corresponding ICD-10 codes, ATC codes, and criminal convictions are shown in Table S1.

***Stratifying variables.*** For analyses stratified by information on the child’s and father’s coresiding, we created a binary variable indicating whether the child and his/her father had lived in the same neighborhood for at least 8 years of the child’s first 10 years of life. Data were obtained from the DeSO (Demographic Statistical Areas) classification, which contains annually updated information on residential areas at the level of county and municipal boundaries in Sweden from 1982 onwards, resulting in a total of 5,984 geographical areas (Statistics Sweden, 2026). Missing information on the place of residence was interpreted as meaning that the child and his/her father lived in a different neighborhood during the year in question.

**Statistical analysis**

***Main analyses.*** For the within-individual Cox regression models, we structured the data as a multiple failure-time panel including all events (failures) between 2003 and 2020 when the child was 10 (or 15) to 30 years old, with each event forming a new observation. We used data from all events that occurred between the ages of 10 (or 15) and 30. We applied a conditional risk-set model with time since last event (or start from study entry) as the underlying time scale, splitting the follow-up time into consecutive time spans. For each individual, the first time span covered the time from study entry (January 1, 2003 or the individual’s 10^th^/15^th^ birthday, whichever occurred later) until the first event. The second and subsequent time spans covered the time from the previous event to either the next event or the end of follow-up, which was the individual’s 30^th^ birthday, emigration, death, or administrative censoring date December 31, 2020, whichever occurred first. Periods of PI were treated as a time-varying exposure within the time spans. This procedure was performed separately for each outcome.

***Age-specific effects.*** To examine potential age-specific effects, we fitted a natural cubic spline with four degrees of freedom for age at exposure, main effect, and interaction with exposure, and then estimated the effect over age in within-individual analyses. The splines had boundary knots at ages 10 and 30, and internal knots at ages 16 and 23. For offspring risk-taking behavior and substance misuse events, the boundary knots were set at ages 15 and 30, and internal knots at ages 19 and 24. We excluded the first time period (time from study entry to the first event) from the analysis to ensure that different underlying time scales would not distort the time-varying estimates.

***Between-individual analyses.*** Standard between-individual Cox regression analyses were conducted for comparative purposes. Separate models were fitted for each outcome, with age as the underlying time scale. In contrast to the within-individual analyses, we only analyzed the time to the first event during follow-up. Individuals were followed up from January 1, 2003, or their 10^th^/15^th^ birthday (15^th^ birthday for substance misuse and risk-taking behavior), whichever was later, until the first occurrence of event, emigration from Sweden (with data from the Migration Register), 30 years of age, death (with data from the Cause of Death Register, Brooke et al., 2017), or the administrative censoring date December 31, 2020, whichever occurred first. The length of follow-up and the child’s age at the start of follow-up thus varied depending on the child’s birth year. Parent’s first prison sentence during follow-up was used as a time-varying exposure, with the timing of exposure determined by the start date of the sentence. Specifically, individuals with an incarcerated parent contributed to the analysis as unexposed until the parent’s first prison sentence, after which the exposure status switched to exposed. The between-individual Cox regression models provided hazard ratios (HR) that estimated the relative risk of the occurrence of each outcome during follow-up in children with an incarcerated father/mother/parent as compared to those without such exposure.

We conducted between-individual analyses with an increasing level of adjustment. First, the models were adjusted for child’s birth year (treated as categorical), father’s and mother’s birth years (categorical), paternal and maternal immigration status (categorical variable defined as being born in Sweden or in another country) from the Total Population Register (Ludvigsson et al., 2016), highest parental education (ordinal variable with four categories ranging from unfinished compulsory education to postgraduate education) from the National Census and the LISA register (Ludvigsson et al., 2019), and parental lifetime psychiatric disorders (dichotomous) from the National Patient Register (Ludvigsson et al., 2011), including diagnoses based on ICD-8 (codes 290–315), ICD-9 (codes 290–319), and ICD-10 (codes F00–F99). In more adjusted models, we included both parents’ criminal convictions (dichotomous variables indicating any paternal/maternal criminal conviction registered between the child’s ages of 10 [or 15] and 30 according to the National Crime Register) and co-parent’s incarceration (dichotomous variable indicating whether the co-parent had been incarcerated between the child’s ages of 10 [or 15] and 30 according to the Sanction Register of the Swedish Prison and Probation Service) as additional covariates.

Regarding the rationale for the chosen covariates, we included child’s birth year to ensure that the results were not influenced by birth cohort effects, as the study sample included several birth cohorts and the follow-up period was long. The adjustment of the incarcerated parent’s birth year controlled for the parent’s age, which is associated with both the likelihood of being incarcerated and offspring outcomes. Adjusting for parental education was intended to account for the effect of socioeconomic background. Immigration status was controlled for as it correlates with many factors (e.g., neighborhood characteristics and socioeconomic factors) that may confound the observed associations. Parents’ lifetime psychiatric disorders were included as covariates to partly adjust for genetic and environmental influences of parental mental health problems on offspring psychiatric and behavioral outcomes. Further adjustments for parental criminal convictions and co-parent’s incarceration were made to better isolate the potential independent effect of parental incarceration from that of related factors.

| **Table S1.** Offspring psychiatric and behavioral events with corresponding ICD-10 codes, Anatomical Therapeutic Chemical (ATC) codes (for psychotropic medication), or convictions. | |
| --- | --- |
|  |  |
| **Event category** | **Description (ICD-10 codes/ATC codes/convictions for offenses)** |
| **Psychiatric disorders** | **ICD-10 codes** |
| Mental and behavioral disorders (excluding mental and behavioral disorders due to psychoactive substance use) | F00–F09, F20–F99 |
| **Substance misuse events** | **ICD-10 codes, convictions for offenses** |
| Mental and behavioral disorders due to psychoactive substance use | F10–F19 |
| Accidental poisoning by and exposure to noxious substances | X40–X49 |
| Poisoning by and exposure to medicaments/biological substances/drugs/alcohol, undetermined intent | Y10–Y15 |
| Convictions for selected traffic offenses | Driving under the influence of alcohol or drugs |
| **Psychotropic medication use** | **ATC codes** |
| Any psychotropic medication | N03AA02, N03AA03, N03AB02, N03AB05, N03AD01, N03AE01, N03AF01, N03AF02, N03AF03, N03AF04, N03AG01, N03AG04, N03AX03, N03AX09, N03AX10, N03AX11, N03AX12, N03AX14, N03AX15, N03AX16, N03AX17, N03AX18, N03AX21, N03AX22, N05AA01, N05AA02, N05AA06, N05AB01, N05AB02, N05AB03, N05AB04, N05AC01, N05AC02, N05AD01, N05AD03, N05AD05, N05AD08, N05AE03, N05AE04, N05AF01, N05AF03, N05AF05, N05AG02, N05AH02, N05AH03, N05AH04, N05AL01, N05AL03, N05AL05, N05AN01, N05AX08, N05AX12, N05AX13, N05BA01, N05BA02, N05BA04, N05BA05, N05BA06, N05BA08, N05BA09, N05BA12, N05BB01, N05BC01, N05BE01, N05CC01, N05CD02, N05CD03, N05CD05, N05CD08, N05CF01, N05CF02, N05CF03, N05CH01, N05CM02, N05CM05, N05CM06, N05CM09, N06AA01, N06AA02, N06AA04, N06AA05, N06AA06, N06AA07, N06AA09, N06AA10, N06AA11, N06AA21, N06AB03, N06AB04, N06AB05, N06AB06, N06AB08, N06AB10, N06AF03, N06AF04, N06AG02, N06AX02, N06AX03, N06AX05, N06AX06, N06AX11, N06AX12, N06AX14, N06AX16, N06AX17, N06AX18, N06AX21, N06AX22, N06AX23, N06BA01, N06BA02, N06BA04, N06BA07, N06BA09, N06BA12, N06BC01, N06BX03, N06BX13, N06DA02, N06DA03, N06DA04, N06DX01 |
| **Risk-taking behavior** | **ICD-10 codes, convictions for offenses** |
| Injury, poisoning, and certain other consequences of external causes | S00–T98 |
| Other external causes of accidental injury | W00–X59 |
| Transport accidents | V00–V99 |
| Convictions for selected traffic offenses | Reckless driving, hit and run offenses, causing death or injury by driving, moving violations |
| **Suicidal behavior** | **ICD-10 codes** |
| Intentional self-harm | X60–X84 |
| Sequelae of intentional self-harm | Y87.0 |
| Event of undetermined intent | Y10–Y34 |
| Sequelae of events of undetermined intent | Y87.2 |

**Table S2.** Convictions for violent and sexual offenses.

| **Offense type** | **Convictions** |  |
| --- | --- | --- |
| Violent crime | Murder, manslaughter, assault, kidnapping, illegal restraint, illegal coercion or threats, robbery, threats or violence against an officer, arson, gross violation of a person’s integrity, harassment | |
| Sexual crime | Rape, sexual coercion, child molestation, sexual intercourse with a child, child pornography offenses, pimping, sexual harassment | |

| **Table S3.** Cumulative incidence (95% confidence intervals) of psychiatric disorders, suicidal behavior, risk-taking, and substance misuse events by age 30 in children with neither parent in prison, father in prison, mother in prison, or either parent in prison between the ages of 10/15 and 30 during the period 2003–2020. | | | | | | | |
| --- | --- | --- | --- | --- | --- | --- | --- |
|  | **Children without parental incarceration** |  | **Children with an incarcerated father** |  | **Children with an incarcerated mother** |  | **Children with either parent incarcerated** |
|  | Cumulative incidence, % |  | Cumulative incidence, % |  | Cumulative incidence, % |  | Cumulative incidence, % |
|  | (95% CI) |  | (95% CI) |  | (95% CI) |  | (95% CI) |
| Psychiatric disorders | 28.3 (28.3–28.4) |  | 46.3 (45.7–46.9) |  | 51.8 (50.2–53.3) |  | 46.7 (46.2–47.3) |
| Psychotropic medication | 43.6 (43.6–43.7) |  | 60.8 (60.3–61.4) |  | 66.5 (65.0–67.9) |  | 61.2 (60.7–61.8) |
| Suicidal behavior | 4.9 (4.9–4.9) |  | 11.0 (10.6–11.4) |  | 14.9 (13.8–16.1) |  | 11.3 (11.0–11.7) |
| Risk-taking behavior | 57.7 (57.6–57.8) |  | 70.2 (69.6–70.7) |  | 73.0 (71.5–74.4) |  | 70.4 (69.8–70.9) |
| Substance misuse events | 9.6 (9.5–9.6) |  | 25.4 (24.8–25.9) |  | 33.1 (31.5–34.8) |  | 26.1 (25.5–26.7) |

# **Figure S1.** Associations between parental incarceration and offspring psychiatric disorders, suicidal behavior, risk-taking, and substance misuse events using within-individual and between-individual designs.

| **Table S4.** Population-level associations between paternal incarceration and offspring psychiatric and behavioral events, stratified by offspring sex, child-father coresiding, paternal offending type, and sentence length. | | | | | | | | | | |
| --- | --- | --- | --- | --- | --- | --- | --- | --- | --- | --- |
|  |  | **Psychiatric disorders** |  | **Psychotropic medication** |  | **Suicidal behavior** |  | **Risk-taking behavior** |  | **Substance misuse events** |
|  |  | HR (95% CI) |  | HR (95% CI) |  | HR (95% CI) |  | HR (95% CI) |  | HR (95% CI) |
| **Stratification by offspring sex** | |  |  |  |  |  |  |  |  |  |
| Male offspring | | 1.18 (1.14–1.21) |  | 1.10 (1.07–1.13) |  | 1.33 (1.25–1.41) |  | 1.09 (1.06–1.12) |  | 1.31 (1.26–1.37) |
| Female offspring | | 1.16 (1.13–1.19) |  | 1.11 (1.08–1.13) |  | 1.16 (1.09–1.23) |  | 1.12 (1.09–1.16) |  | 1.26 (1.20–1.33) |
| **Stratification by child-father coresiding** | |  |  |  |  |  |  |  |  |  |
| Living in the same neighborhood | | 1.18 (1.12–1.22) |  | 1.08 (1.04–1.12) |  | 1.23 (1.12–1.34) |  | 1.07 (1.03–1.11) |  | 1.34 (1.25–1.43) |
| Living in a different neighborhood | | 1.08 (1.05–1.11) |  | 1.04 (1.01–1.06) |  | 1.15 (1.09–1.21) |  | 1.08 (1.05–1.11) |  | 1.20 (1.15–1.24) |
| **Stratification by paternal offending type** | | |  |  |  |  |  |  |  |  |
| Father with violent offending | | 1.09 (1.05–1.12) |  | 1.04 (1.01–1.07) |  | 1.08 (1.01–1.15) |  | 1.06 (1.02–1.09) |  | 1.13 (1.07–1.19) |
| Father without violent offending | | 1.12 (1.08–1.15) |  | 1.07 (1.04–1.09) |  | 1.18 (1.11–1.25) |  | 1.07 (1.05–1.10) |  | 1.27 (1.21–1.32) |
| **Stratification by sentence length** | |  |  |  |  |  |  |  |  |  |
| Sentences of less than 1 year | | 1.11 (1.09–1.14) |  | 1.11 (1.09–1.13) |  | 1.20 (1.15–1.25) |  | 1.06 (1.04–1.08) |  | 1.28 (1.24–1.32) |
| Sentences of 1 year or more | | 1.13 (1.10–1.17) |  | 1.15 (1.11–1.18) |  | 1.19 (1.11–1.27) |  | 1.10 (1.07–1.14) |  | 1.27 (1.20–1.34) |
| All models were adjusted for child's and parents' birth years, parents' immigration status, highest parental education, parents' psychiatric disorders, parents' criminal convictions, and co-parent's incarceration during the follow-up. Missing information on parental education and immigration status were treated as separate categories.  HR = hazard ratio; CI = confidence interval | | | | | | | | | | |

| **Table S5.** Cox regression models comparing the risk of offspring events during and after the first exposure to paternal incarceration, relative to the pre-exposure period. | | | | |
| --- | --- | --- | --- | --- |
|  |  | **Stratified Cox regression** (within-individual) | | |
|  |  | First exposure to PI vs. pre-exposure period |  | Post-exposure period vs. pre-exposure period |
|  |  | HR (95% CI) |  | HR (95% CI) |
| Psychiatric disorders | | 1.08 (1.00–1.17) |  | 0.84 (0.77–0.92) |
| Psychotropic medication | | 1.10 (1.04–1.16) |  | 0.94 (0.88–0.99) |
| Suicidal behavior | | 0.92 (0.68–1.25) |  | 0.42 (0.28–0.64) |
| Risk-taking behavior | | 1.10 (1.01–1.21) |  | 0.75 (0.67–0.83) |
| Substance misuse events | | 1.05 (0.88–1.25) |  | 0.84 (0.69–1.02) |
| Pre-exposure period is used as the reference category in both comparisons. Both the pre-exposure and post-exposure periods are limited to a maximum of one year.  HR = hazard ratio; CI = confidence interval | | | | |

| **Table S6.** Associations between paternal incarceration and offspring psychiatric and behavioral events using within-individual design, with the first time span for each individual (i.e. time span from study entry to the first event) included vs. excluded from the analysis. | | | | |
| --- | --- | --- | --- | --- |
|  |  | **Stratified Cox regression**  (within-individual) | | |
|  |  | All observations included |  | Observations before the first event excluded |
|  |  | HR (95% CI) |  | HR (95% CI) |
| Psychiatric disorders | | 1.00 (0.96–1.04) |  | 1.00 (0.96–1.04) |
| Psychotropic medication | | 1.02 (0.99–1.05) |  | 1.01 (0.98–1.04) |
| Suicidal behavior | | 1.08 (0.93–1.25) |  | 1.14 (0.95–1.36) |
| Risk-taking behavior | | 1.01 (0.97–1.06) |  | 1.00 (0.95–1.05) |
| Substance misuse events | | 0.99 (0.91–1.06) |  | 1.01 (0.93–1.10) |
| All models were adjusted for age as a linear effect.  HR = hazard ratio; CI = confidence interval | | | | |

| **Table S7.** Associations between paternal incarceration and offspring psychiatric and behavioral events, with analysis restricted to diagnoses received during unplanned health care visits. | | | | |
| --- | --- | --- | --- | --- |
|  |  | **Stratified Cox regression**  (within-individual) | | |
|  |  | All diagnoses included |  | Diagnoses set during unplanned health care visits included |
|  |  | HR (95% CI) |  | HR (95% CI) |
| Psychiatric disorders | | 1.00 (0.96–1.04) |  | 0.99 (0.85–1.16) |
| Psychotropic medication | | – |  | – |
| Suicidal behavior | | 1.08 (0.93–1.25) |  | 0.98 (0.69–1.39) |
| Risk-taking behavior | | 1.01 (0.97–1.06) |  | 1.02 (0.95–1.09) |
| Substance misuse events | | 0.99 (0.91–1.06) |  | 0.90 (0.81–1.02) |
| All models were adjusted for age as a linear effect.  HR = hazard ratio; CI = confidence interval | | |  |  |

| **Table S8.** Paternal incarceration and offspring psychiatric and behavioral events using within-individual design with different age adjustments. | | | | |
| --- | --- | --- | --- | --- |
|  |  | **Model 1** |  | **Model 2** |
|  |  | HR (95% CI) |  | HR (95% CI) |
| Psychiatric disorders | | 1.00 (0.96–1.04) |  | 1.00 (0.96–1.04) |
| Psychotropic medication | | 1.02 (0.99–1.05) |  | 1.02 (1.00–1.05) |
| Suicidal behavior | | 1.08 (0.93–1.25) |  | 1.08 (0.93–1.25) |
| Risk-taking behavior | | 1.01 (0.97–1.06) |  | 1.01 (0.97–1.06) |
| Substance misuse events | | 0.99 (0.91–1.06) |  | 0.99 (0.92–1.07) |
| Model 1: Adjusted for age as a linear effect. | | | | |
| Model 2: Adjusted for age as a natural cubic spline with three degrees of freedom. | | | | |
| HR = hazard ratio; CI = confidence interval | | | | |

# **References**

Brottsförebyggande rådet (2025). *Swedish crime statistics.* Retrieved from <https://bra.se/bra-in-english/home-html>

Brooke, H. L., Talback, M., Hornblad, J., Johansson, L. A., Ludvigsson, J. F., Druid, H., Feychting, M., & Ljung, R. (2017). The Swedish cause of death register. *European Journal of Epidemiology*, *32*, 765–773.

Ludvigsson, J. F., Almqvist, C., Bonamy, A. K. E., Ljung, R., Michaëlsson, K., Neovius, M., Stephansson, O., & Ye, W. M. (2016). Registers of the Swedish total population and their use in medical research. *European Journal of Epidemiology*, *31*, 125–136.

Ludvigsson, J. F., Andersson, E., Ekbom, A., Feychting, M., Kim, J. L., Reuterwall, C., Heurgren, M., & Olausson, P. O. (2011). External review and validation of the Swedish national inpatient register. *BMC Public Health*, *11*, 450.

Ludvigsson, J. F., Svedberg, P., Olén, O., Bruze, G., & Neovius, M. (2019). The longitudinal integrated database for health insurance and labour market studies (LISA) and its use in medical research. *European Journal of Epidemiology*, *34*, 423–437.

Statistics Sweden (2026). *DeSO – Demografiska statistikområden*. Retrieved from <https://www.scb.se/hitta-statistik/regional-statistik-och-kartor/regionala-indelningar/demografiska-statistikomraden-deso/>

Wallerstedt, S. M., Wettermark, B., & Hoffmann, M. (2016). The first decade with the Swedish prescribed drug register – A systematic review of the output in the scientific literature. *Basic & Clinical Pharmacology & Toxicology*, *119*, 464–469.
